# Ice formation and its elimination in cryopreservation of oocytes

**DOI:** 10.21203/rs.3.rs-4144933/v1

**Published:** 2024-05-23

**Authors:** Abdallah W. Abdelhady, David W. Mittan-Moreau, Patrick L. Crane, Matthew J. McLeod, Soon Hon Cheong, Robert E. Thorne

**Affiliations:** a Department of Clinical Sciences, College of Veterinary Medicine, Cornell University, Ithaca, NY 14853; b Molecular Biophysics and Integrated Bioimaging Division, Lawrence Berkeley National Laboratory, Berkeley, CA 94720; c Physics Department, Cornell University, Ithaca, NY 14853; d MiTeGen, LLC, Ithaca, NY 14850

**Keywords:** cryopreservation, oocyte, vitrification, ice formation, assisted reproduction, x-ray diffraction

## Abstract

Damage from ice and potential toxicity of ice-inhibiting cryoprotective agents (CPAs) are key issues in assisted reproduction of humans, domestic and research animals, and endangered species using cryopreserved oocytes and embryos. The nature of ice formed in bovine oocytes (similar in size to oocytes of humans and most other mammals) after rapid cooling and during rapid warming were examined using synchrotron-based time-resolved x-ray diffraction. Using cooling rates, warming rates and CPA concentrations of current practice, oocytes show no ice after cooling but always develop large ice fractions — consistent with crystallization of most free water — during warming, so most ice-related damage must occur during warming. The detailed behavior of ice at warming depended on the nature of ice formed during cooling. Increasing cooling rates allows oocytes soaked as in current practice to remain essentially ice free during both cooling and warming. Much larger convective warming rates are demonstrated and will allow routine ice-free cryopreservation with smaller CPA concentrations. These results clarify the roles of cooling, warming, and CPA concentration in generating ice in oocytes and establish the structure and grain size of ice formed. Ice formation can be eliminated as a factor affecting post-thaw oocyte viability and development in many species, improving outcomes and allowing other deleterious effects of the cryopreservation cycle to be independently studied.

## Introduction

Effective cryopreservation^1–3^ of oocytes^4–6^ and embryos^7^ is critical for routine assisted reproduction of humans^8^ and of domestic^7,9–11^, research^12,13^, and endangered^14–17^ animals, and for long-term preservation of valuable genetics. Overall reproductive success rates using frozen oocytes and embryos relative to unfrozen controls vary widely between species and can vary widely between laboratories and practitioners.

The mechanisms by which cryopreservation protocols damage cells and degrade reproductive outcomes are incompletely understood, but many are related to intracellular ice formation and its inhibition. Ice nucleate when the sample temperature drops below the equilibrium melting temperature *T*_*m*_, and the nucleation rate becomes very large near the homogeneous nucleation temperature (*T*_*h*_ ~ 137− °C in pure water)^18,19^. Ice crystal growth rates in aqueous cryoprotective agents (CPAs) solutions peak at temperatures between *T*_*h*_ and *T*_*m*_, and become negligible at cryogenic temperatures^20^. To inhibit intracellular ice formation, oocytes/embryos are soaked in equilibration and vitrification solutions containing increasing concentrations of penetrating (e.g., ethylene glycol (EG), dimethyl sulfoxide (DMSO)) and non-penetrating (e.g., sucrose) CPAs to reduce the “free” (non-hydration) water^21^ concentration in the cell, cooled to cryogenic temperature, and stored. To return the oocyte to a physiological state, the oocyte must be warmed then gradually rehydrated to allow the penetrating CPAs to leave the cell. The degree of ice formation depends on the extent of dehydration which is limited by the concentration of penetrating CPAs used. Penetrating CPAs are toxic, especially at high concentrations^22–25^ thus necessitating a compromise between protection from ice and toxicity.

Ice crystals formed inside cells may puncture membranes and disrupt the spindle apparatus, cytoskeleton, and other cellular structures. Ice crystals are nearly pure water, so their growth concentrates proteins, other cytoplasmic solutes, and CPAs in remaining uncrystallized solution, which may lead to protein aggregation and denaturation and increase CPA toxicity. Physicochemical properties including solubility, pH, pKas of amino acids, and the hydrophobic effect are all temperature dependent^26^. Even without ice formation, these may cause conformational changes, partial unfolding, and aggregation of proteins^27^ and changes in structure of other cellular components, some of which may be irreversible. Ice formation and differential contraction of sample regions during cooling can cause sample fracturing — especially in larger samples — that disrupts membranes and other cellular structures.^28–31^

Experiments by Seki, Mazur and coworkers suggested that warming is more important than cooling in determining oocyte and embryo survival and reproductive outcomes^32–37^, and this has been supported by subsequent experiments^38,39^. During warming, water molecules initially locked in a vitreous state become increasingly mobile. Pre-existing crystallites can grow, new crystals can nucleate and grow, and larger crystals can grow at the expense of smaller ones (recrystallization).

Despite the assumed central role of ice in determining cryopreservation outcomes, remarkably little has been done to characterize ice formed during the fast-cooling cryopreservation cycle. Ice within cells can be visually detected as darkness or milkiness. This optical assay is neither sensitive nor quantitative, especially when applied to cells (e.g., bovine oocytes) with dark, lipid-rich cytoplasm that prevents visualization of internal structures. Cryocooled cells can be examined by transmission electron microscopy and ice crystals directly imaged^40^. Rates of ice formation and amounts of ice formed during cooling and warming can be assessed using calorimetry^41–43^ if cooling and warming rates are sufficiently small. Studying ice formation in oocytes during rapid warming is particularly challenging as observations must be made during the short warming transient, requiring time resolutions <<100 ms.

Synchrotron-based x-ray diffraction provides a powerful and quantitative approach to studying ice in cold samples^44,45^. X-rays easily penetrate through lipid-rich bovine oocytes, which are similar in size and x-ray absorption coefficient to protein crystals used in biomolecular crystallography^46,47^. X-ray diffraction can detect ice volume fractions well below 1% and the total ice diffraction flux is proportional to the amount of ice present. X-ray diffraction has been widely used to study ice formation in other contexts, including in aqueous cryoprotectant solutions and in protein crystals^44,48^. X-ray diffraction has been used to observe ice in cryocooled embryos, cumulus-oocyte complexes, and morulae^45^, but has not been systematically applied.

Here we use static and time-resolved x-ray diffraction to characterize the amount, structure, and grain size of ice formed during cooling and warming within bovine ooctyes, which are similar in size and thermal properties to most mammalian oocytes^49^. Using cooling rates, warming rates and CPA concentrations comparable to those in current practice, nearly all “free” internal water crystallizes during warming, even when no ice is detected after cooling. Using much larger cooling rates achieved using tools from cryocrystallography, ice formation during both cooling and warming is largely eliminated. Initial efforts at optimizing convective warming yields warming rates a factor of ~20 larger than in current practice, which will allow CPA concentrations for ice free cryopreservation to be reduced. A critical factor affecting post-thaw oocyte viability and development can now be eliminated, and other deleterious effects of the cryopreservation cycle can be independently studied. Similarities with the bovine model^50^ suggest that our methods should be directly applicable to cryopreservation of human oocytes and embryos.

## Results

**Figure 1.**
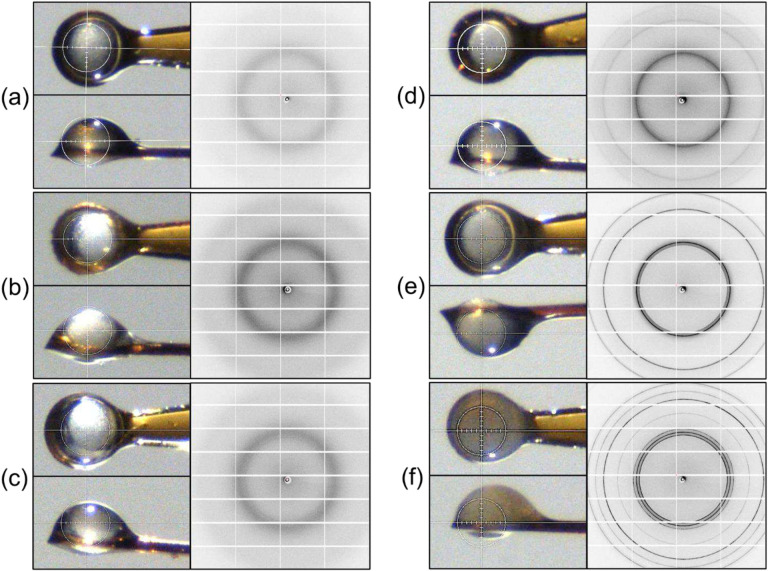
Correlation between optical images and diffraction patterns of cryocooled bovine oocytes at *T*=−173°C. **(a)** Oocyte soaked in 100% strength vitrification solution (15% DMSO, 15% EG., 0.5 M sucrose) and fast cooled in LN_2_, as described in the text, showing two diffuse rings characteristic of low-density amorphous ice *I*_LDA_. **(b)** Oocyte soaked in 50% vitrification solution and fast cooled. Diffuse scattering characteristic of *I*_LDA_. **(c)** Oocyte soaked in 90% vitrification solution and slow cooled in cold N_2_ gas before plunging in LN_2_. Diffuse but somewhat sharper scattering, largely consistent with *I*_LDA_. **(d)** Oocyte soaked in 80% vitrification solution and slow cooled, showing well defined but somewhat broad diffraction rings at resolutions expected for cubic ice *I*_c_. **(e)** Oocyte soaked in 40% vitrification solution and fast cooled. Sharp diffraction rings at cubic ice resolutions and beginning of hexagonal ice ring. **(f)** Oocyte soaked in 40% vitrification solution and slow cooled. Strong and azimuthally inhomogeneous rings at all expected resolutions of hexagonal ice *I*_h_. White circle in each oocyte image has a diameter of 100 μm.

Oocytes were placed either on crystallography supports or on Cryotop^™^ supports (Figs 1 and **S1**) popular in assisted reproduction and cooled at ~30,000 °C/min (typical of current practice) or ~600,000 °C/min (when using crystallography supports) in liquid nitrogen (LN_2_) using an automated, liquid-nitrogen-based cryocrystallography instrument **(Figs. S2, S3)**. During x-ray data collection, these samples were kept cold using a *T*=−173 °C N_2_ gas stream (**Fig. S4**). To warm the samples *in situ*, the *T*=−173 °C gas stream was blocked using an air blade shutter and a room temperature N_2_ gas stream directed at the sample (**Fig. S4**).

### Ice diffraction and structure after cooling.

**Figure 2.**
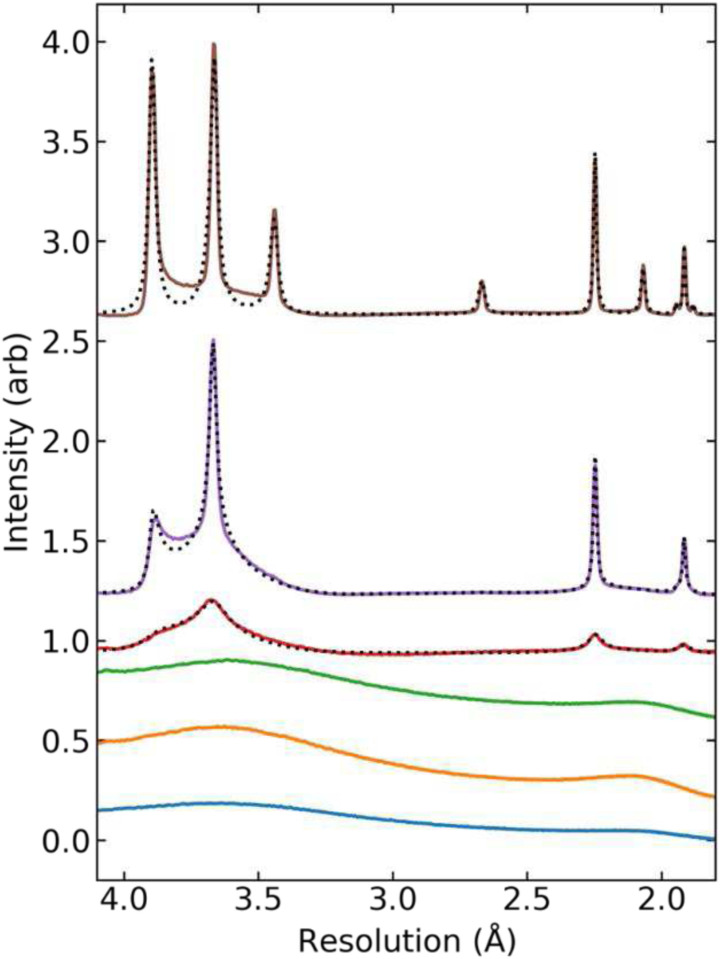
Variation of ice diffraction intensity versus resolution with amount and type of ice. Azimuthally integrated and background subtracted diffraction intensity corresponding to the samples and x-ray detector images shown in Figure 1 (a)–(f), arranged from bottom to top. The dotted black lines for the top three are fits to a model of stacking disordered ice with hexagonal plane stacking fractions *Φ*_h_ of **(d)** 0.35, **(e)** 0.40, and **(f)** 0.72.

Observed 2D diffraction patterns from bovine oocytes at *T*=−173 °C vary with CPA concentration and cooling rate, as shown in Figs. 1, 2 and **S5**. The patterns are of five basic types:

1. two broad, diffuse and azimuthally uniform rings, one near 3.7 Å and a weaker ring near 2.2 Å, consistent with low-density amorphous ice *I*_*LDA*_;
2. three broad, diffuse and azimuthally uniform rings at expected cubic ice *I*_*c*_ resolutions, consistent with a small-grain powder of stacking disordered ice *I*_*sd*_^51,52^ (**Sec. S7**, **Fig. S6**) with a small hexagonal plane fraction *Φ*_h_;
3. four or more narrower, azimuthally continuous and “smooth” rings at resolutions expected for *I*_*c*_ and *I*_*h*_, consistent with a small grain powder of *I*_*sd*_ with a larger hexagonal plane fraction *Φ*_h_;
4. sharp, continuous rings with significant azimuthal variation, consistent with largely hexagonal ice *I*_*h*_ and the presence of some larger grains; and
5. multiple discrete peaks on top of weaker, azimuthally continuous but nonuniform rings, indicating the presence of a modest number of even larger *I*_*h*_, crystals (**Fig. S5**).

For all CPA concentrations and cooling rates used here, diffraction as in (5) was observed only when an error allowed partial thawing and then refreezing of the sample. Measurements using oocytes that were moved through oil before mounting and cooling (**Sec. S2**) confirmed that observed diffraction arose from ice inside the oocyte and not on its surface.

Fig. 2 shows diffraction intensity versus resolution, obtained from 2D diffraction patterns as in Fig. 1 by azimuthal integration and background subtraction. The black dotted lines are best fits to a model of stacking disordered ice^52^ with the hexagonal stacking fractions *Φ*_h_ indicated (**Sec. S7, Fig. S6**). The quality of such fits is generally excellent for all oocytes examined, particularly when the cubic stacking fraction *Φ*_**c**_ **=**1-*Φ*_h_ is substantial. Deviations between data and fit may be due in part to background modeling and due to sample inhomogeneity (e.g., the presence of ice crystals with a distribution of *Φ*_h_ values and crystal sizes.)

### Correlation between optical images at T=−173 °C and x-ray diffraction.

Optical images (Fig. 1) were acquired using the beamline camera (**Fig. S4**). When x-ray diffraction showed no evidence of crystalline ice, oocytes showed internal “grains” with an opalescent character, associated with their high lipid content. When x-ray diffraction showed largely “cubic” ice (as in Fig. 1(d)), optical images were largely indistinguishable from those of fully vitrified oocytes. When x-ray diffraction indicated the presence of largely hexagonal ice (Fig. 1(f)), the oocytes had a milky / cloudy appearance that completely obscured the oocyte’s internal structure. X-ray diffraction produces substantial ice signals even when ice crystallites are too small or sparse to generate strong optical scattering.

### Effects of CPA concentration and cooling rate on ice formed during cooling.

Cryoprotectant concentrations required to obtain plunge-cooled oocytes with no visible crystalline ice diffraction decrease with increasing cooling rate. For oocytes soaked in 100% strength vitrification solution (see Methods), mounted on crystallography loops (Figs. 1, **S1(a)**) and cooled at ~30,000 °C/min, no ice diffraction was observed. For oocytes soaked in 80% vitrification solution, the diffraction pattern (Fig. 1(d)) consisted of continuous and azimuthally uniform ice rings, consistent with powder-like ice, at the resolutions of *I*_c_. Fits to intensity versus resolution plots indicate that the ice was stacking disordered *I*_*sd*_ with a large cubic fraction.

For oocytes on crystallography loops cooled at ~600,000 °C/min, ice-free diffraction patterns were obtained even when soaked in 50% vitrification solution (Fig. 1(b)) — corresponding to the standard equilibration solution. For 40% strength, azimuthally uniform ice rings, consistent with very small grain size ice *I*_sd_ with a large cubic fraction, were observed (Fig. 1(e)). The factor of two reduction in minimum CPA concentration for a factor of ~20 increase in cooling rate is consistent with the behavior of drops of single CPA solutions, for which the minimum CPA concentration varies logarithmically with inverse cooling rate^53^.

For oocytes mounted on thin Cryotops^™^ (**Fig. S1(b)**) and cooled using plunge cooler settings that gave the largest cooling rates, no ice was observed for 90% strength vitrification solution, and diffuse, largely cubic *I*_*s*d_ was observed at 80% strength. Oocytes on the thicker Cryotops (**Fig. S1(c)**) showed strong, sharp diffraction consistent with largely hexagonal *I*_sd_ at 80% strength. Cooling rates achieved using current sample supports are substantially lower than those using cryocrystallography supports under otherwise identical conditions.

In all these experiments, as CPA concentrations were decreased, ice diffraction patterns followed the progression shown in Fig. 1 from *I*_*sd*_ with a large cubic fraction to *I*_*sd*_ with a small cubic fraction to *I*_*h*_.

### Estimates and x-ray diffraction measurement of warming rates.

Oocytes were thawed *in situ* at the x-ray beamline using a room temperature N_2_ gas stream while diffraction data was recorded (**Fig. S4**). Oocyte warming rates were estimated from 12 fps videos recorded using the beamline camera, from evolution of x-ray diffraction patterns and from evolution of the ice unit cell volume (**Sec. S9**). For oocytes on crystallography supports that developed ice during cooling, ice diffraction began evolving ~0.02 s after the solenoid controlling the air blade and warming gas stream flows opened and disappeared 0.08 to 0.12 s later. This gives an average warming rate between the cold gas stream temperature (−173 °C) and 0 °C of ≈100,000 °C/min. With the room-temperature gas stream shut off to allow warming in stagnant air, all ice diffraction disappeared after ~0.42 s, corresponding to an average warming rate of ~24,000 °C/min.

For oocytes on thin Cryotops (**Fig. S1(b)**), ice diffraction disappeared after 0.22–0.31 s in the warm gas stream, corresponding to an average warming rate of ~40,000 °C/min. For oocytes on thick, curved Cryotops (**Fig. S1(c)**), ice disappeared after 0.5–0.6 s, corresponding to an average warming rate of 19,000 °C/min. These are factors of ~2.5 and ~5 smaller than are achieved on crystallography loops. The physics of heat transfer in room-temperature N_2_ gas and in LN_2_ at its boiling temperature are similar. Cryotop cooling rates should thus also be ~2.5 and ~5 times smaller, consistent with the larger minimum CPA concentrations they require to prevent ice formation on cooling.

**Figure 3.**
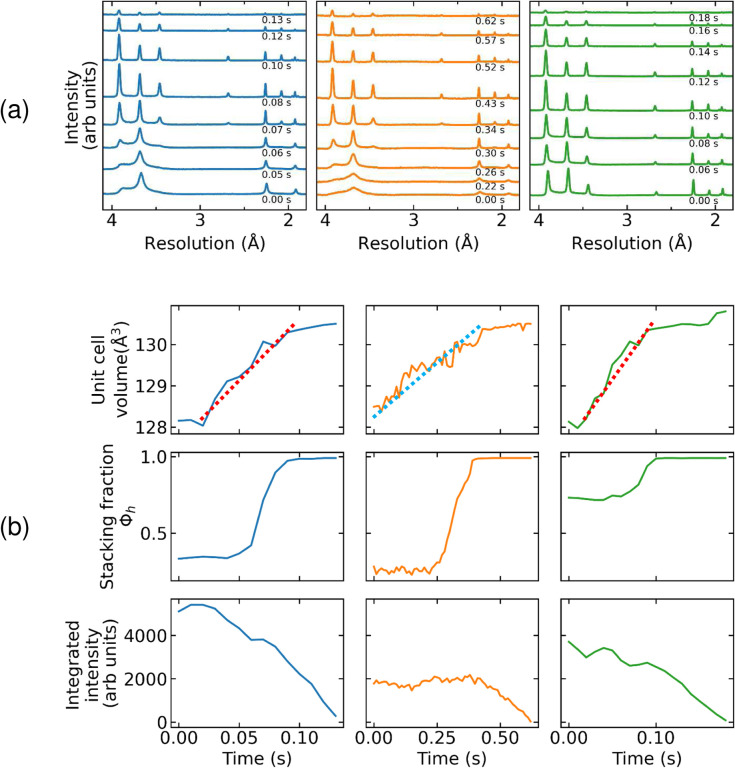
Analysis of ice diffraction during warming. **(a)** Azimuthally integrated and background subtracted diffraction intensity versus resolution and **(b)** equivalent hexagonal ice unit cell volume, hexagonal stacking fraction *Φ*_h_, and integrated diffraction intensity in ice versus time during warming of bovine oocytes. Samples from left to right: oocyte on a crystallography support, soaked in 40% vitrification solution and fast cooled; oocyte on a thick Cryotop, soaked in 60% vitrification solution and fast cooled; and oocyte on a crystallography support, soaked in 40% vitrification solution and slow cooled. Parameters were extracted from DIFFaX fits to azimuthally integrated and background subtracted diffraction patterns. Time *t*=0 corresponds to the opening of the valve controlling room temperature N2 gas flow. Sample warming, as indicated by a change in unit cell parameter, began ~20 ms after valve opening, due to the time required to establish warm gas flow at the sample position. Unit cell volumes of ~128.1 Å^3^ and ~130.5 Å^3^ correspond to temperatures of ~100 K and ~273 K (**Fig. S7**). Dotted red lines in the upper left and right panels of **(b)** have a slope corresponding to a warming rate of ~130,000 °C/min. The dotted turquoise line in the upper middle panel of **(b)** corresponds to a warming rate of 25,000 °C/min.

Oocyte warming rates can be more accurately estimated from the resolution (2θ) values of ice rings vs time (**Sec. S9.2**). These values give the equivalent hexagonal unit cell volume of ice, whose temperature dependence has been accurately determined (**Fig. S7**),^54^ providing a direct, non-contact way of measuring the temperature inside the cell. Azimuthally integrated and background subtracted diffraction patterns (Fig. 3(a)) were fit to a model of stacking disordered ice, yielding both the ice unit cell volume and the hexagonal stacking fraction *Φ*_h_ versus time (Fig. 3(b).) Average warming rates between ~ −173 °C and ~ 0 °C for oocytes on crystallography supports were typically ~ 130,000 °C/min, and maximum observed rates (from the slope of unit cell versus time) were ~ 200,000 °C/min. These warming rates are larger than the largest reported using Cryotops of 117,000 °C/min^33^ and larger than “typical” values of 42,000 °C/min^55^. This is surprising: Our rates are for warming in a room temperature N_2_ gas stream, whereas the largest previously reported rates are for warming in a ~37 °C thawing solution that has far better heat transfer properties.

### Ice diffraction and structure during warming.

Almost all oocytes — regardless of cooling rate, CPA concentrations, sample support type and warming gas stream velocity — developed crystalline ice during warming. The detailed behavior depended on the nature of any ice formed during cooling.

For oocytes showing no evidence of ice after cooling, diffraction progressed from the appearance and sharpening of diffuse rings at cubic ice resolutions, to appearance of additional rings at resolutions of hexagonal ice, to fade-out and disappearance of all ice diffraction. In no case did an oocyte that was ice diffraction-free after cooling develop discrete ice diffraction peaks or significant azimuthal inhomogeneity in its ice rings. The x-ray illuminated volume thus contained a very large number of crystallites and the average ice crystal size remained very small throughout warming. Ice ring intensity first became significant near −70 °C, increased until ~ −20 °C, and disappeared near 0°C (with temperatures directly determined from ice diffraction.) When using crystallography supports, the time interval from appearance to disappearance of ice rings was typically 40–80 ms. These quantitative observations during rapid warming can be compared with observations of mouse oocyte “blackening” in fixed temperature and slow warming experiments^56^.

Fig. 3 **(a)** shows examples of azimuthally integrated diffraction intensity versus resolution measured during warming of bovine oocytes that exhibited ice diffraction after cooling. Fig. 3 **(b)** shows the equivalent hexagonal ice unit cell volume, hexagonal stacking fraction *Φ*_h_, and integrated diffraction intensity in ice versus time deduced from DIFFaX fits to the data in **(a)**. For oocytes that exhibit rings primarily at cubic ice resolutions (Fig. 3 left and middle samples; **Fig. S8**) before warming, no significant change is observed until the sample temperature reaches ~ −90 to −70 °C. As warming continues, additional rings at resolutions unique to hexagonal ice appear and the rings develop significant azimuthal variations in intensity, suggesting growth of larger crystals. *Φ*_h_ begins increasing at −90 to −70 °C and reaches a value of 1.0, indicating that ice has evolved to pure *I*_h_, at ~ −20 °C. The time from the start of ice ring evolution to ring disappearance was typically 100–130 ms. The integrated intensity in ice rings increased modestly (e.g., by 20%) or remained constant even as ice transformed from “cubic” with *Φ*_h_~0.3–4 to hexagonal with *Φ*_h_ =1, before decreasing at later times.

For oocytes with ice rings at all hexagonal positions and *Φ*_h_ >0.8 after cooling (Fig. 3, right sample), the integrated intensity in ice rings and thus the amount of ice initially present was large and did not increase appreciably during warming, suggesting that all crystallizable water had crystallized during cooling. As the oocyte warmed above ~ −70 °C, the azimuthal “lumpiness” of the rings increased and the diffracted intensity in the brightest spots grew modestly (generally by less than ~50%), indicating growth of modestly larger ice crystals. Larger crystallites correlated with a longer time required for all ice to finally melt: the time from the start of ice ring evolution to disappearance of the brightest spots was typically 140–220 ms. Similar behavior during warming was observed for oocytes on CryoTops (**Figs. S9, S10**).

### Estimates of ice crystallite sizes.

As discussed in **Sec. S11**, for the cooling rates, warming rates, and CPA concentrations examined here, average ice grain sizes within bovine oocytes cooled on crystallography supports are small compared with the ~500 nm — 1 μm thought (based on limited evidence from a few cell types) to be large enough to cause mechanical cell damage^40,57^. Exceptions occurred when oocytes were improperly cooled or were accidentally thawed and refrozen at the beamline (e.g., **Fig. S5**). We cannot deduce the distribution of ice grain sizes within oocytes (**Sec. S11**), although it does not appear to have a significant large-size tail. There may still be “black swans” — ice crystals that, based on a measured distribution, are improbably large^58^ — that could play an outsize role in determining whether an oocyte survives and thrives. These may be associated with heterogeneity within the oocyte, e.g., cellular compartments with different internal (including CPA) compositions, and interior regions disrupted by handling,

### CPA concentration dependence of total ice diffraction intensity and “free” water.

**Figure 4.**
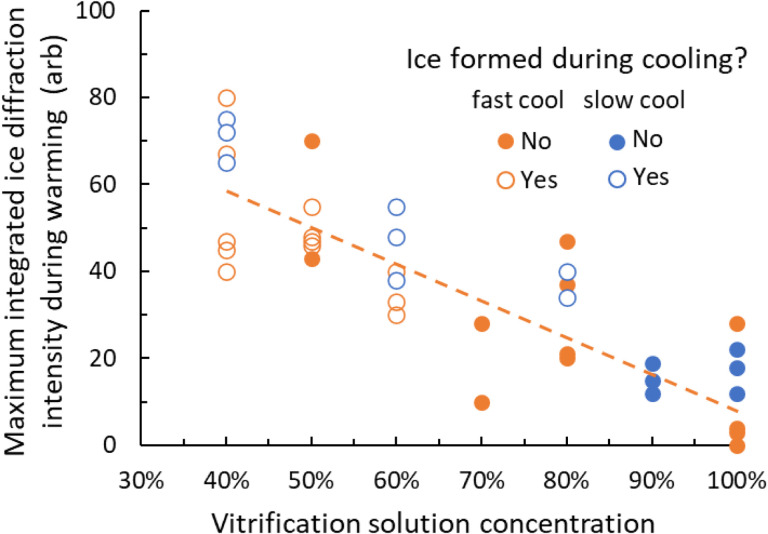
Effect of CPA concentration and cooling rate on amount of ice formed in bovine oocytes during warming. Maximum integrated intensity in ice diffraction rings observed during warming versus CPA concentration in the vitrification solution used. Oocytes were slow cooled (at ~30,000 °C/min, orange circles) or fast cooled (at ~600,000 °C/min, blue circles), and then warmed at ~150,000 °C/min in a N_2_ gas stream. Closed and open symbols indicate oocytes that did and did not show crystalline ice diffraction after cooling and before warming. The dotted line is a fit to the fast cooled sample data. Experimental scatter may reflect variations in the initial state/quality of the oocytes, in CPA concentration within oocytes introduced in the soaks, or in the (small) amount of surface liquid (in which ice is more likely to nucleate and grow).

The total diffraction in all ice rings is proportional to the total amount of ice present within the X-ray illuminated volume and thus to the ice volume fraction within the oocyte. As shown in Fig. 4, the maximum total diffraction in ice rings observed during warming increases roughly linearly with decreasing CPA concentration. As CPA concentration decreases, the amount of “free”, non-hydration water available for crystallization increases^21^ and maximum ice fractions should increase accordingly. Assuming additivity of the effects of EG and DMSO, data in Ref. 21 give free water fractions (by weight) in 100% and 40% strength vitrification solutions of ~50% and ~80%, respectively. Within the oocyte, proteins and other solutes are present in total concentrations of the same order as those in protein crystals, where 50% or more of water may be bound and unavailable for crystallization^44^. Consequently, the amount of free water within an oocyte soaked in 100% vitrification solution may be close to zero, consistent with the absence of ice formation during both fast and slow cooling. Maximum total diffraction intensities observed (vs time and CPA concentration) during warming were comparable to that observed for a “snowball” oocyte (**Fig. S5**)(accidently thawed and then slowly cooled in the T=−173 °C gas stream at the x-ray beamline). This indicates that at nearly all CPA concentrations, most of the crystallizable water did in fact crystallize before final melting.

### Cooling rate dependence of maximum ice fractions during warming.

Maximum ice diffraction intensities and ice fractions observed during warming appear to be independent of cooling rate — for the cooling rates examined here — at lower CPA concentrations (Fig. 4). At higher CPA concentrations where most samples showed no ice after cooling, the smallest maximum ice diffraction intensities during warming were observed for the largest cooling rates. Ice observed during warming is due to ice formed both on cooling and during warming. The number of ice nuclei formed during cooling is proportional to the cooling time, which is inversely proportional to the cooling rate. Our observations suggest that if sufficient ice nuclei are present after cooling (due to slower cooling and/or lower CPA concentrations), ice grows so rapidly during warming that the maximum ice fraction is largely determined by the available crystallizable water^44^. This is true even when nuclei present after cooling do not yield visible ice diffraction.

### Ice-free cryopreservation of bovine oocytes.

Ice nucleation and growth rates during cooling and warming decrease as CPA concentration increases. With adequate CPA concentrations, sufficiently fast cooling to minimize ice nuclei formed, and sufficiently fast warming to limit growth of those nuclei and nucleation and growth of additional nuclei, completing the cryopreservation cycle of cooling and warming without formation of detectable ice should be possible.

**Figure 5.**
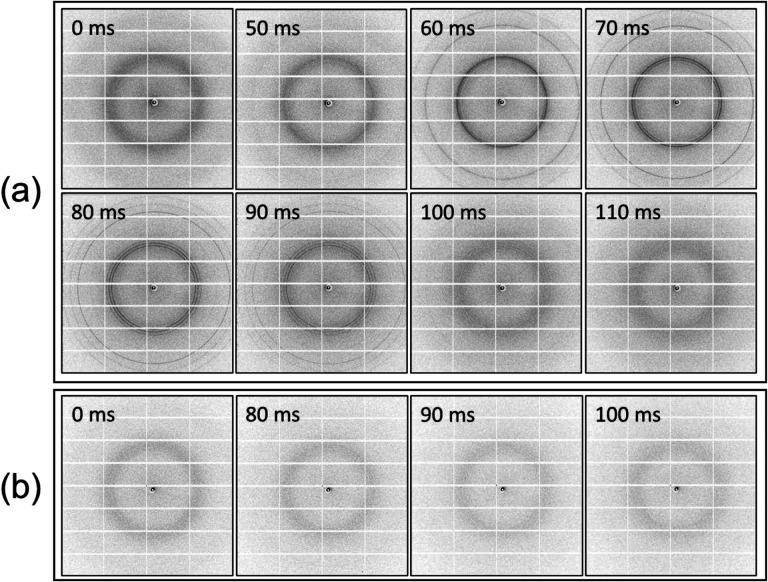
Evolution of oocyte diffraction during warming. Time series of diffraction images acquired during warming of **(a)** the oocyte of Fig. 1(b), soaked in 50% vitrification solution, moved through oil to remove surface solvent, and “fast” cooled; and **(b)** the oocyte of Fig. 1(a), soaked in 100% vitrification solution and “fast” cooled. In **(a)**, ice diffraction becomes visible at 50 ms, evolves from diffuse rings at cubic ice resolutions to sharp rings at cubic ice locations at 60 ms to rings at all hexagonal ice locations at 80 ms, and has disappeared at 110 ms. In **(b)**, only one partial, extremely weak ice diffraction ring is observed, most likely arising from surrounding solvent, and for less than 20 ms.. **Fig. S11** shows corresponding intensity vs resolution plots with DIFFaX fits.

Fig. 5(a) shows diffraction during warming at ~150,000 °C/min from an oocyte that was soaked in 50% strength vitrification solution and cooled at ~600,000 °C/min on a crystallography support. No ice diffraction is evident after cooling, and ice rings that form during warming remain continuous and homogeneous. Fig. 5(b) shows diffraction during warming at ~150,000 °C/min from an oocyte that was soaked in 100% strength vitrification solution and cooled at ~600,000 °C/min on a crystallography support. In this case, ice diffraction that appeared during warming was extremely weak and visible for only ~15 ms. This tiny amount of ice (**Fig. S11**) may have formed in residual surface liquid rather than in the solute-rich oocyte interior; extracellular ice formation typically does not affect subsequent cell survival^40,59^. As discussed below, convective warming in a thawing solution (rather than in a gas stream) should give warming rates an order of magnitude larger and allow CPA concentrations required for an ice-free freeze-thaw cycle to be reduced.

## Discussion

### Ice formation during cooling and warming.

The progression of ice diffraction patterns and ice crystal form in cryocooled bovine oocytes as CPA concentration is increased — from pure hexagonal ice *I*_*h*_ to stacking disordered ice *I*_*sd*_ with increasing cubic character to low-density amorphous ice *I*_LDA_ — follows the general sequence observed vs CPA concentration in protein crystals^44^. The reverse progression is observed in time when initially ice-free oocytes are warmed. Ice diffraction patterns from oocytes exhibit asymmetric and peak/resolution dependent broadening (Figs. 2 and **S6**) that is poorly fit if a mix of cubic and hexagonal crystals is assumed, but that is accurately reproduced by a model of stacking disordered ice^52^. For pure water, stacking disorder arises when ice growth occurs under conditions of deep supercooling or from amorphous ice^60^, as is expected to arise during cooling and warming at rates achieved here.

CPA concentrations, cooling rates, and sample supports used in current assisted reproduction practice appear adequate to give cold bovine oocytes that are largely ice free. During warming at rates comparable to the largest reported using these supports^33^, these same samples develop a substantial volume fraction of crystalline ice. The grain size of this ice — reflected by the azimuthal nonuniformity of ice diffraction rings — remains small through to melting. The impact of this transiently formed ice on post-thaw viability and development will be reported elsewhere, but the small average grain size makes large mechanical impacts less probable. This is consistent with observations that, compared with “fresh” oocytes not subject to cryopreservation protocols, the fraction of cryopreserved bovine oocytes that survive is modestly reduced, although developmental outcomes are more severely affected.

Using the same sample supports and cooling protocols, reducing CPA concentrations by ~20% or more leads to ice formation on cooling and to more nonuniform ice diffraction and larger grain size ice during warming. Since EG and DMSO, used together with sucrose in standard vitrification solutions, are efficient in inhibiting ice formation, finding alternative CPA combinations that are as or more effective in preventing ice formation at lower concentrations may be hard. Time-consuming soaking protocols to minimize osmotic shock will remain necessary when using current sample supports and cooling and warming protocols.

The relatively small increases in azimuthal lumpiness of ice rings observed during warming for nearly all samples studied here suggest that ice crystal growth via recrystallization was modest. This is consistent with the short times (~30 ms when using crystallography supports, and 75–150 ms using Cryotops) for ice growth in our experiments, and with modest ice growth velocities expected in the presence of substantial concentrations of CPAs and of native solutes (e.g., proteins) in the oocyte cytoplasm.

### Tools from cryocrystallography allow large reduction of CPA concentrations.

Ice formation has long been an issue in x-ray cryocrystallography of biomolecules. The typical size range of biomolecular crystals — from a few micrometers to a few hundred micrometers — includes the size range of mammalian oocytes. The typical range of crystal solvent contents — from ~30% to ~80% v/v — includes that of oocytes (before CPA soaks and dehydration). Tools to hold and cool crystals have been optimized over the last 30 years to maximize cooling rates, and the cooling instrument used here has allowed thin biomolecule-containing films on cryo-EM supports to be fully vitrified (requiring cooling rates >1.2×10^7^ °C/min) using boiling nitrogen^61^. Application of these tools to bovine oocytes allows ice-free diffraction to be obtained from cold samples even when using 50% strength vitrification solution. The cooling rates achieved of ~600,000 °C/min are ~20 times larger than the 23,000 °C/min reported when using Cryotops^62,63^ and the ~35,000 °C/min obtained using thin wall quartz microcapillaries^64–66^. Use of “slushed” nitrogen (cooled to its melting temperature) increased cooling rates in quartz capillaries to ~250,000 °C/min^64^. Use of slushed nitrogen in (suitably modified) cryocrystallography cooling instruments should increase cooling rates for bovine (and similar size) oocytes to ~2×10^6^ °C/min. Cryocrystallography tools are not optimized for routine cryopreservation practice but they demonstrate what should be routinely achievable.

Successful cryoprotectant-free cryopreservation of mouse fibroblast cells by ink-jet printing ~40 pL drops onto a cryocooled substrate has been reported^67^. Ice likely formed in the drops during cooling and certainly formed during warming (**Sec. S13**). Post-thaw cell viability approaching 90% is then likely due to tolerance of the chosen cells to small ice crystals. Bovine and human oocytes have much larger volumes (~500 pL) than the fibroblast cells (~5 pL) and drops used, maximum achievable cooling and warming rates are lower, and successful CPA- and dehydration-free cryopreservation seems unlikely. Complete elimination of CPAs may in any case be undesirable because they modulate thermal expansion^68^ and so can reduce stress and fracturing.

### Ice formation during cooling *and* warming can be eliminated.

Using cryocrystography tools and a room-temperature N_2_ gas stream for warming, essentially no ice ever forms in oocytes soaked in standard vitrification solution. Previous claims of ice-free oocyte cryopreservation have not been supported by direct measurements, let alone by an ice detection measurement as sensitive and quantitative as X-ray diffraction.

Warming rates directly measured here of 130,000 °C/min (average between −196 °C and 0 °C) and 200,000 °C /min (maximum) for bovine oocytes on crystallography supports in a room temperature N_2_ gas stream are comparable to the largest warming rates (117,000 °C /min) reported for 50 μm thermocouples attached to Cryotops and plunged into thawing solutions^33^. Warming rates measured here in stagnant air of ~24,000 °C/min are only 4–5 times smaller than those reported in thawing solutions. This is surprising. Aqueous thawing solutions have heat transfer properties that are vastly superior to those of N_2_ gas; they are also superior to those of LN_2_ and are predicted^69^ and measured^34^ to give correspondingly larger warming rates.

With proper protocol optimization, plunging ~120 μm oocytes on suitably low-thermal-mass supports into warm (37 °C) thawing solutions should give warming rates of ~10^6^ °C/min. **Fig. S12** shows results of our initial attempts to optimize convective (plunge) warming in a standard 37 °C thawing solution. Warming rates between −140 °C and −20 °C of 1.4 ×10^6^ °C /min and 1.6 ×10^7^ °C /min are recorded for 125 μm and 25 μm junction (80 μm and 25 μm wire) thermocouples, respectively. When combined with cooling rates demonstrated here and those feasible using slushed nitrogen, ice formation within oocytes in the freeze-thaw cycle should be completely and routinely eliminated using standard vitrification solution concentrations and perhaps even using 50% strength solutions.

Alternative warming methods — such as using lasers to heat samples containing or coated with a strong absorber (e.g., carbon black, gold nanorods)^36,70,71^ — can give larger warming rates (~10^7^ °C/min from simulations) and have particular potential for achieving fast warming of large samples including tissues and organs^38^. This approach requires substantial technical development to be practical in routine assisted reproduction practice, and CPA concentrations to avoid ice formation (roughly proportional to the logarithm of the warming rate^72^) will be only modestly reduced relative to what can be achieved with optimized convective warming. For oocytes and embryos with minimum dimensions below ~500 μm, optimized convective warming will be preferred.

### Warming and cooling rates are both important.

As previously noted^35–37^, much of the earlier cryopreservation literature focuses on the importance of cooling rates in determining outcomes. An alternative suggestion, based on experiments demonstrating the importance of warming rates to mouse oocyte viability, is that cooling rates are unimportant as long as warming is fast enough^35–37^. While theoretically true, this is not a sound basis for optimization.

The critical warming rate (CWR) required for no ice formation in a nominally ice-free sample decreases as cooling rate increases beyond the critical cooling rate (CCR)^72^. At the CCR, the ice fraction in the sample will be below a detection limit — perhaps 0.1% with X-ray diffraction, larger when using other methods. As cooling rate is increased beyond the CCR, the number of ice nuclei and ice fraction will be reduced in inverse proportion but will still be finite, providing seeds for growth during warming. As the number density of these seeds drops, the “fast enough” warming rate required to keep maximum ice fractions below a target value drops. For optimal cryopreservation, both cooling and warming rates should be maximized.

### Summary and future prospects.

Ice formation during cooling and warming has long been considered a key issue in cryopreservation of oocytes and embryos for assisted reproduction. Synchrotron-based x-ray diffraction provides a robust, straightforward, and high-throughput method to characterize ice inside oocytes and how its formation and properties depend on factors including cooling rate, warming rate, and CPA concentration. Substantial ice is observed during warming even in samples that show no ice diffraction after cooling and, in all cases, the largest ice diffraction intensities and ice grain sizes are observed during warming. Using tools from cryocrystallography, bovine oocytes can be cooled and warmed sufficiently fast so that no ice ever forms, and significant further increases in both cooling and warming rates and reductions in CPA concentrations required for ice-free cryopreservation are feasible.

To the extent that ice formation, CPA toxicity, and osmotic stress are important for oocytes from a given species, the present results and methods promise to yield substantial improvements in post-thaw survival and developmental outcomes. They will allow ice formation to be ruled out as a cause of differences in outcomes between cryopreserved and fresh oocytes, allowing other damage mechanisms to be identified. The principles and methods demonstrated here should be broadly applicable in optimizing cryopreservation of all small biological samples.

## Materials and Methods

### Oocyte preparation and cryopreservation.

Denuded MII stage bovine oocytes were prepared as described in **Sec. S2**. Standard equilibration solution (ES) used in cryoprotection of bovine oocytes contain 7.5% DMSO and 7.5% EG and standard vitrification solution (VS) contains 15% v/v DMSO, 15% v/v EG, and 0.5 M sucrose. Oocytes were soaked in ES and VS concentrations ranging from 40% to 100% of these standard values, as described in **Sec. S3**.

Immediately after the vitrification solution soak, oocytes were placed with as little surrounding vitrification solution as possible onto one of three polymer sample supports **(Fig. S1)**: a 10 μm thick cryocrystallography sample support with a 100 μm aperture (MicroLoop LD 100, MiTeGen, Ithaca, NY), a flat, ~90 μm thick Cryotop (Kitazako, Japan), commonly used in human and animal oocyte and embryo cryopreservation, or a curved, 200 μm thick Cryotop. To facilitate handling at the synchrotron, the sample supporting end of each Cryotop was removed and inserted into a cryocrystallography goniometer base. To verify that observed ice diffraction originated from within the oocyte, some were translated through a low-viscosity oil to remove aqueous surface solution.

Oocytes on supports were immediately loaded into an automated cryocooling instrument developed for cryocrystallography (**Sec. S4**, **Fig. S2**), plunged into liquid nitrogen (LN_2_) at 77 K, and automatically loaded into 16-sample cryocrystallography “pucks” (**Fig. S3**). The automated cryocooler can be operated in several modes that give different cooling rates. Cooling rates in “fast” mode for 25 μm junction bead thermocouples are >3,000,000 °C/min. Cooling rates for 100 μm diameter bovine oocytes on cryocrystallography supports are estimated to be ~600,000 °C /min in “fast” mode and ~30,000 °C /min in “slow” mode (**Sec. S4**). The “slow” value is comparable to the largest “open system” cooling rates (i.e., where the ooycte/embryo directly contacts LN_2_) achieved in routine IVF practice^55^ and roughly half the cooling rate of ~69,000 °C/min^34^ reported for a 50 μm thermocouple attached to a Cryotop.

### X-ray data collection and oocyte warming.

X-ray diffraction data was collected from a total of 179 bovine oocytes at MacCHESS beamline ID7B2 at the Cornell High-Energy Synchrotron Source (CHESS) using the experimental setup shown in **Fig. S4**, as described in **Sec. S5**. A 12.8 keV x-ray beam focused to a 10 × 10 μm (FWHM) spot was directed through the oocyte center, and x-ray diffraction was recorded using a Pilatus 6M detector framing at 100 Hz, giving 10 ms time resolution. Oocytes were cooled by a *T*=100 K (−196 °C) dry N_2_ gas stream, and warmed by simultaneously interrupting the cold stream using a room temperature N_2_ gas “air blade” and initiating a flow of room temperature N_2_ gas at the sample.

### Processing and modelling of ice diffraction.

Diffraction patterns captured by an area detector record the scattered x-ray intensity at different angles 2*θ* relative to the incident x-ray beam direction, which by Bragg’s law correspond to different resolutions *d*=*λ*/2sin(*θ*).

Processing and modeling of detector frames and ice diffraction largely followed the protocol used by Moreau et al.^44^ in a study of ice diffraction from protein crystals. Detector frames were processed to fit and remove background and azimuthally integrated (**Sec. S6**). Diffraction intensity versus resolution plots for each frame from each oocyte were analyzed (**Sec. S7)** by embedding the program DIFFaX^73^, which calculates diffraction from samples containing stacking faults, in an optimization routine to determine best-fit parameters for stacking disordered ice formed of planes of hexagonal (*I*_*h*_) and cubic (*I*_*c*_) ice. This fitting procedure yielded four parameters for each frame / time point: the hexagonal stacking fraction *Φ*_*h*_, the lattice constants *a* and *c* of the equivalent hexagonal unit cell, the overall scale of the intensity, and an instrumental broadening parameter. These were then plotted versus frame number / time.

## Data Availability

Raw diffraction frame sets will be deposited in the XRDa Crystal Raw Data Archive (xrda.pdbj.org). Beamline images of samples, processed diffraction data with fits, and plots of parameters extracted from diffraction data will be archived in Cornell’s Open Science Framework instance, https://osf.io/institutions/cornell.
